# Melatonin antagonizes interleukin‐18‐mediated inhibition on neural stem cell proliferation and differentiation

**DOI:** 10.1111/jcmm.13140

**Published:** 2017-04-21

**Authors:** Zheng Li, Xingye Li, Matthew T.V. Chan, William Ka Kei Wu, DunXian Tan, Jianxiong Shen

**Affiliations:** ^1^ Department of Orthopaedic Surgery Peking Union Medical College Hospital Chinese Academy of Medical Sciences and Peking Union Medical College Beijing China; ^2^ Department of Anaesthesia and Intensive Care The Chinese University of Hong Kong Hong Kong China; ^3^ State Key Laboratory of Digestive Disease LKS Institute of Health Sciences The Chinese University of Hong Kong Hong Kong China; ^4^ Department of Cellular and Structural Biology Health Science Center University of Texas San Antonio TX USA

**Keywords:** neural stem cells, melatonin, interleukin‐18, spinal cord injury

## Abstract

Neural stem cells (NSCs) are self‐renewing, pluripotent and undifferentiated cells which have the potential to differentiate into neurons, oligodendrocytes and astrocytes. NSC therapy for tissue regeneration, thus, gains popularity. However, the low survivals rate of the transplanted cell impedes its utilities. In this study, we tested whether melatonin, a potent antioxidant, could promote the NSC proliferation and neuronal differentiation, especially, in the presence of the pro‐inflammatory cytokine interleukin‐18 (IL‐18). Our results showed that melatonin *per se* indeed exhibited beneficial effects on NSCs and IL‐18 inhibited NSC proliferation, neurosphere formation and their differentiation into neurons. All inhibitory effects of IL‐18 on NSCs were significantly reduced by melatonin treatment. Moreover, melatonin application increased the production of both brain‐derived and glial cell‐derived neurotrophic factors (BDNF, GDNF) in IL‐18‐stimulated NSCs. It was observed that inhibition of BDNF or GDNF hindered the protective effects of melatonin on NSCs. A potentially protective mechanism of melatonin on the inhibition of NSC's differentiation caused IL‐18 may attribute to the up‐regulation of these two major neurotrophic factors, BNDF and GNDF. The findings indicate that melatonin may play an important role promoting the survival of NSCs in neuroinflammatory diseases.

## Introduction

Spinal cord injury can be resulted from neural trauma, inflammation or degeneration, and it is a serious clinical disorder. The patients suffer from their immobility, and it also poses enormous economic burden to their family and also to the society 1, 2, 3, 4. It is estimated that the incidence of spinal cord injury is 40–80 cases of per million yearly and there are around 273,000 patients with spinal cord injury in USA alone 5, 6, 7. NSCs are pluripotent, undifferentiated and self‐renewing neuronal precursor cells. NSC's transplantation has been promulgated as a promisingly attractive strategy for spinal cord injury and neurodegenerative disorders 8, 9, 10, 11. NSC can replace lost oligodendrocytes and neurons, and this replacement contributes to the restoration of motor function in animal models 12, 13, 14, 15. However, low survival rate of transplanted NSCs is a major obstacle to the success of this therapy 16, 17, 18. It was reported that when the NSCs were transplanted into spinal cord, their vitality and proliferation were impaired 2, 19, 20. This impairment is at least partially mediated by the release of pro‐inflammatory cytokines. These cytokines will induce cellular apoptosis, axonal destruction and extensive demyelination during secondary spinal cord injury 21.

Cytokine IL‐18 is a member of the IL‐1 family 22. It is produced by a variety of cells, including dendritic cells, adipocytes and macrophages 23, 24, and it is also detectable in the ependymal cells, activated microglia, astrocytes, neurons and the pituitary gland. Increasing evidence has confirmed that IL‐18 is a pleiotropic cytokine, which regulates both humoral and cellular immunity and plays an important role in the inflammatory cascade 25. Previous studies have demonstrated that IL‐18 mediated astrocyte–microglia interactions in the spinal cord to aggravate neuropathic pain after neural injury 26. On other hand, neural injury also induced IL‐18 expression in the dorsal horn 27. These reactions form a vicious cycle and manifest the neural injury furtherly. IL‐18 also has the capacity to inhibit neuronal survival and differentiation on cultured NSCs 28.

Melatonin (*N*‐acetyl‐5‐methoxytryptamine), synthesized from the pineal gland as well as in the peripheral tissues, plays the important roles in various physiological processes including the circadian rhythm, reproduction and the cerebrovascular and neuroimmuno‐endocrine functions 29, 30. Melatonin also exerts neuroprotective effects in various pathological conditions of the central nervous system including Alzheimer's disease, Parkinson's disease, ischaemic brain injury and spinal cord injury 31, 32, 33. Recently, several studies showed that melatonin enhanced growth of NSCs and their differentiation into neurons 32, 34, 35. However, it is unknown whether melatonin could promote NSC's proliferation, survival or differentiation under the inflammatory conditions. Herein, we examined the effect of melatonin on the proliferation and differentiation of NSCs in the presence of IL‐18.

## Materials and methods

### Cell culture and reagents

All experimental protocols had been approved by the Clinical Research Ethics Committee of the Peking Union Medical College Hospital. NSCs were obtained from 13.5‐day‐old embryos of Wistar rats using an established method 36. In brief, the telencephalon was separated under a stereotaxic microscope and dissected into small pieces. NSCs were released by incubation of these dissected brain tissues with 0.25% trypsin and kept in neurobasal medium (Hyclone, Logan, UT, USA) with 2% B27 (Invitrogen, USA), basic fibroblast growth factor (bFGF; Invitrogen, Carlsbad, CA, USA), epidermal growth factor (Invitrogen) and their inhibitor (TrkB‐Fc and MAB212, respectively) at 37°C under a humidified atmosphere containing 5% CO_2_. Melatonin, IL‐18 and luzindole were obtained from Sigma‐Aldrich. Cells were treated with or without IL‐18 (1–100 ng/ml) in the presence of melatonin (10 ng/ml) and/or luzindole (5 μM). All mouse experiments are carried out in accordance with the relevant institutional and national guidelines and regulations, approved by the Animal Care and Use Committee of Peking Union Medical College Hospital and conform to the relevant regulatory standards.

### Immunofluorescence

Cells were fixed with 3.5% formaldehyde, permeabilized with 0.1% Triton X‐100, blocked with 3% BSA and 0.05% Tween‐20 in PBS and then incubated with primary antibody(dilutions 1:5000) (Nestin, β‐tubulin III and glial fibrillary acidic protein (GFAP); Sigma‐Aldrich, Oakville, ON, Canada) overnight at 4°C. Nuclei were stained with 4, 6‐diamidino‐2‐phenylindole (DAPI) (Sigma‐Aldrich). Fluorescence was measured by a confocal microscope (TCS SP2, Leica, Mannheim, Germany).

### Western blots

Total protein was extracted from cells and separated using 10% sodium dodecyl sulphate‐polyacrylamide gel electrophoresis gel. Proteins were transferred onto a nitrocellulose membrane (Millipore, MA, USA), and the membrane was blocked with non‐fat milk. Then, the membrane was probed with primary antibodies (dilutions 1:5000) (Sigma‐Aldrich) and bound with horseradish peroxidase‐conjugated secondary antibodies. Chemiluminescent signal was developed using the ECL kit (Millipore, MA, USA).

### Enzyme‐linked immunosorbent assay

Enzyme‐linked immunosorbent assay (ELISA) was performed to measure brain‐derived neurotrophic factor (BDNF) and glial cell‐derived neurotrophic factor (GDNF) levels in the supernatant of cell culture according to the manufacturer's instructions (R&D Systems, MN, USA). The absorbance was measured at a 450‐nm wavelength.

### Cell proliferation

Cell proliferation was measured using a Cell Counting Kit‐8 (CCK‐8, Dojindo, Kumamoto, Japan) according to the manufacturer's instructions. The cells were cultured for 0, 24, 48 or 72 hrs, and the absorbance was measured at a 450‐nm wavelength.

### RNA isolation and reverse transcription‐quantitative PCR (qPCR)

Total RNA was extracted using Trizol Reagent (Invitrogen) from cells following to the manufacturer's instructions. PCR was performed with specific primers in 20 μl PCR mixtures for 35 cycles. The levels of mRNA were measured by SYBR Green quantitative PCR performed on the iQ5 Real‐Time PCR Detection System (Bio‐Rad, Hercules, CA, USA). The primers used for PCR amplification were shown in Table 1. Quantification data were normalized to GAPDH.

**Table 1:** Primer sequence

Name	Sequence (5′–3′)
BDGF	CCTCCTCTGCTCTTTCTGC
	TGGGATTACACTTGGTCTCGT
GDNF	TCTGCCTGGTGTTGCTCC
	CCTCTGCGACCTTTCCCT
GAPDH	AATGGGCAGCCGTTAGGAAA
	TGAAGGGGTCATTGATGGCA
MT1	AGTGTCATTGGCTCGGTAT
	GCTTCAGTTTGGGTTTGCT
MT2	ACCCTTACCCACTCATCCTT
	TCTCAGCCTTTGCCTTCCTT
β‐tubulin‐III	AGCAAGGTGCGTGAGGAGTA
	AAGCCGGGCATGAAGAAGT

### Statistics analysis

Data were expressed as means ± standard deviation (SD). Student's *t*‐test was performed for comparison between two groups, and an analysis of variance (anova) was used to compare multiple groups and followed by Student's *t*‐test for comparison between two groups. *P* < 0.05 were considered statistically significant.

## Results

**Figure 1:**
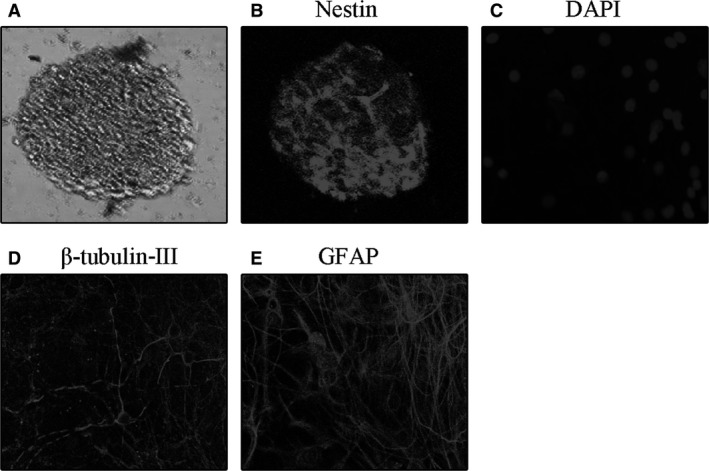
The images of Proliferation and differentiation of NSCs. (**A**) Representative photomicrograph of neurospheres in NSC culture. (**B**) Immunofluorescence staining for nestin of purified NSCs. (**C**) Nucleus staining with DAPI of differentiated cells derived from NSCs. (**D**) Immunofluorescence staining for β‐tubulin‐III‐positive neurons derived from NSCs. (**E**) Immunofluorescence staining for GFAP‐positive protoplasmic astrocytes derived from NSCs.

The cells cultured in neurobasal medium supplemented with bFGF and B27 proliferated and formed neurospheres on the second day after culture (Fig. 1A and B). These neurospheres also expressed the NSC‐specific marker, nestin (Fig. 1C). Three days after bFGF withdraw, these neurospheres differentiated into neurons and astrocytes (Fig. 1D and E). The results confirmed that the isolated cells were NSCs.

**Figure 2:**
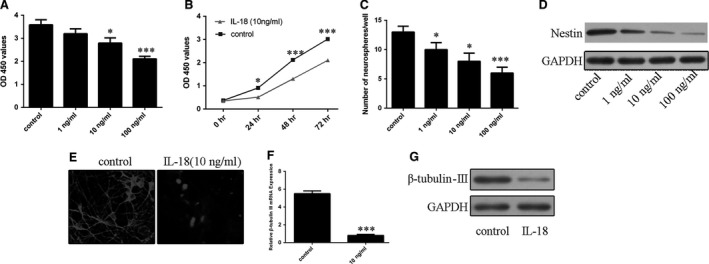
Effects of IL‐18 on NSC proliferation and differentiation. (**A**) The proliferation of NSCs incubated with increasing concentrations of IL‐18 (1–100 ng/ml) was measured by CCK‐8 assay. (**B**) The time‐dependent effect of IL‐18 (10 ng/ml) on NSC proliferation was measured by CCK‐8 assay. (**C**) IL‐18 concentration‐dependent neurosphere formation. (**D**) Nestin protein expression was detected by Western blots. (**E**) Effects of IL‐18 (10 ng/ml) on β‐tubulin‐III‐positive cells in NSC culture. (**F**) Effects of IL‐18 (10 ng/ml) on β‐tubulin‐III mRNA expression. (**G**) Effects of IL‐18 (10 ng/ml) on β‐tubulin‐III protein expression. *P < 0.05 and ***P < 0.001.

CCK‐8 assay showed that IL‐18 (1–100 ng/ml) significantly repressed NSC proliferation in a dose‐dependent manner (Fig. 2A). In addition, time‐dependent inhibition of NSC proliferation by IL‐18 (10 ng/ml) was observed (Fig. 2B). Moreover, IL‐18 decreased neurosphere formation (Fig. 2C) accompanied with inhibition of nestin expression (Fig. 2D). NSCs incubation with IL‐18 for 3 days also significantly decreased β‐tubulin‐III‐positive cells as confirmed by immunofluorescence (Fig. 2E). qRT‐PCR and Western blot analyses showed that IL‐18 inhibited the β‐tubulin‐III mRNA and protein expression (Fig. 2F and G).

**Figure 3:**
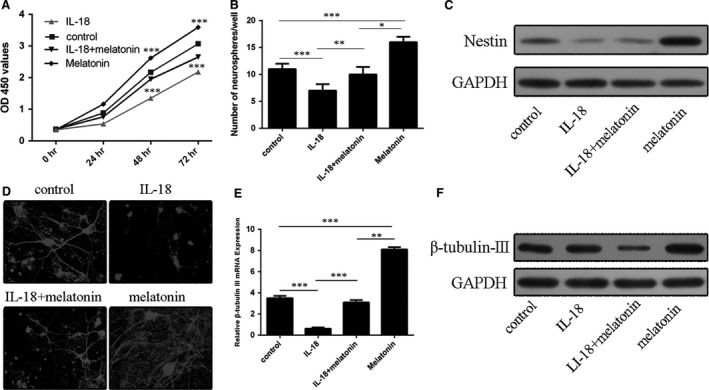
Effects of melatonin on the NSC proliferation and differentiation. (**A**) The proliferation of NSCs incubated with or without IL‐18 (10 ng/ml) in the presence or absence of melatonin (10 ng/ml). (**B**) Melatonin increased neurosphere formation in the presence or absence of IL‐18 and melatonin. (**C**) Nestin expression in IL‐18‐challenged NSCs with or without melatonin. (**D**) Immunofluorescence staining for β‐tubulin‐III‐positive neurons derived from NSCs incubated with or without IL‐18 (10 ng/ml) and melatonin (10 ng/ml). (**E**–**F**) Effects of melatonin on β‐tubulin‐III (**E**) mRNA and (**F**) protein expression in IL‐18‐challenged NSCs. *P < 0.05; **P < 0.01; ***P < 0.001.

The results indicate that melatonin *per se* had a profound effect to promote NSCs proliferation. When melatonin co‐incubated with IL‐18, it partially reversed the inhibitory effect of IL‐18 on NSCs (Fig. 3A). Moreover, similar protective effects of melatonin on neurosphere formation in the absence or presence of IL‐18 were observed (Fig. 3B). Western blot analysis found that melatonin up‐regulated basal nestin expression in NSCs and partially reversed the reduced nestin level caused by IL‐18 (Fig. 3C). In addition, NSCs treated with melatonin for 3 days significantly restored β‐tubulin‐III‐positivity levels of NSCs which suppressed by IL‐18 (Fig. 3D). qRT‐PCR and Western blot analyses also showed that melatonin up‐regulated the expression of β‐tubulin‐III mRNA and its protein while it abrogated the inhibitive effects of IL‐18 on them in cultured NSCs (Fig. 3E and F).

**Figure 4:**
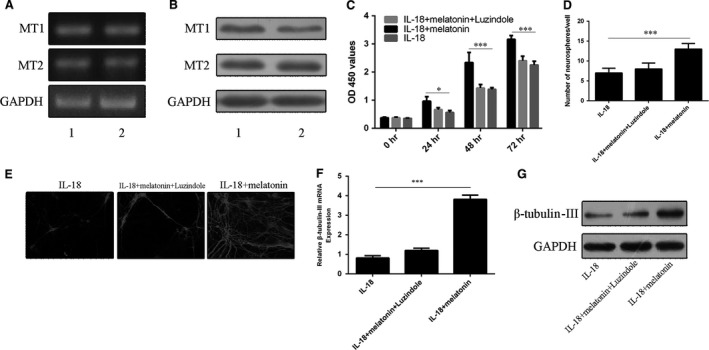
Effects of melatonin and its receptor antagonist luzindole on proliferation and differentiation of NSCs under IL‐18 challenging. (**A**) mRNA and (**B**) protein expression of melatonin receptors of MT1 and MT2, respectively. (**C**) The proliferation of NSCs treated with IL‐18 and/or melatonin (10 ng/ml) in the absence or presence of luzindole (5 μM). (**D**) Effects of luzindole on neurosphere formation with or without IL‐18 and/or melatonin. (**E**) Immunofluorescence staining for β‐tubulin‐III‐positive neurons derived from NSCs treated as above. (**F**) mRNA and (**G**) protein levels of β‐tubulin‐III. *P < 0.05; ***P < 0.001.

RT‐PCR and Western blot analyses confirmed that mRNA and protein of melatonin receptors (MT1 and MT2) were expressed in NSCs (Fig. 4A and B). However, the protective effects of melatonin on NSCs mentioned above were blocked by co‐incubated with luzindole (Fig. 4C and D). This suggests that the melatonin receptors may be involved in the protective effects of melatonin on the NSC suppression mediated by IL‐18 (Fig. 4C and D). Likewise, luzindole abolished the promoting effect of melatonin on β‐tubulin‐III positivity (Fig. 4E) as well as β‐tubulin‐III mRNA (Fig. 4F) and protein (Fig. 4G) expression in IL‐18‐challenged NSCs.

**Figure 5:**
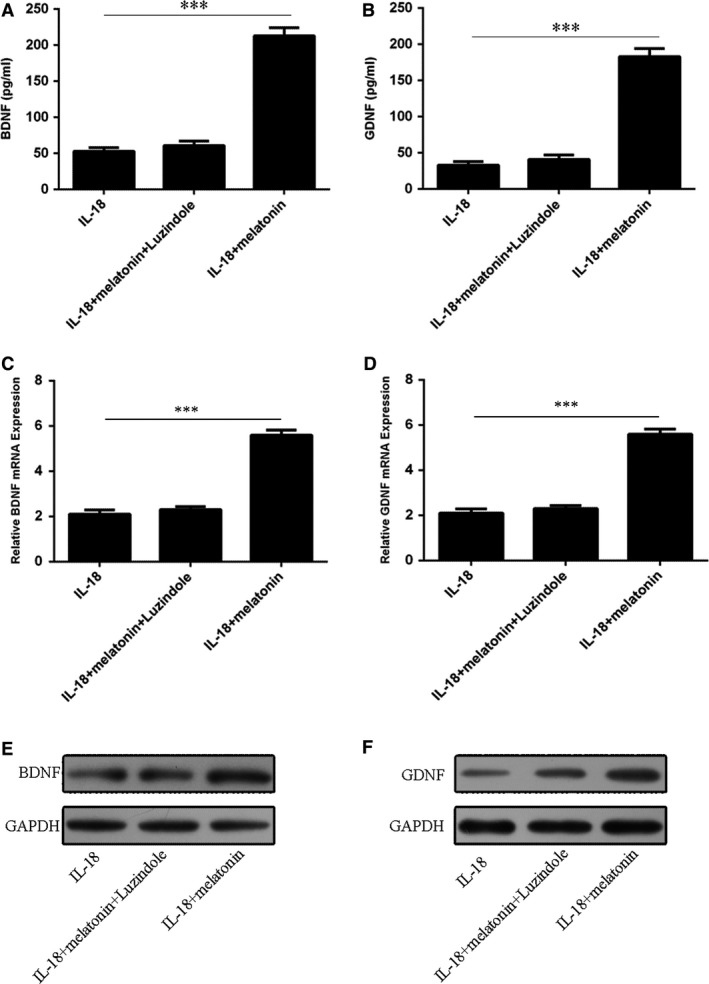
Effects of melatonin on BDNF and GDNF contents of NSCs treated with IL‐18. (**A**) BDNF and (**B**) GDNF contents, (**C**–**D**) The mRNA level of BDNF (**C**) and mRNA level GDNF (**D**) in NSCs treated with or without IL‐18 and/or melatonin (10 ng/ml) in the presence or absence of luzindole (5 μM). (**E**–**F**) The protein levels of BDNF and GDNF. ***P < 0.001.

BDNF and GDNF are two important growth factors involved in NSC survival and proliferation. In this regard, ELISA results showed that melatonin drastically increased contents of both BDNF and GDNF and these were also blocked by luzindole (Fig. 5A and B). Similarly, qRT‐PCR and Western blot analyses found that melatonin increased mRNA and protein expressions of both BDNF and GDNF while luzindole co‐incubation abrogated these up‐regulations (Fig. 5C–F).

**Figure 6:**
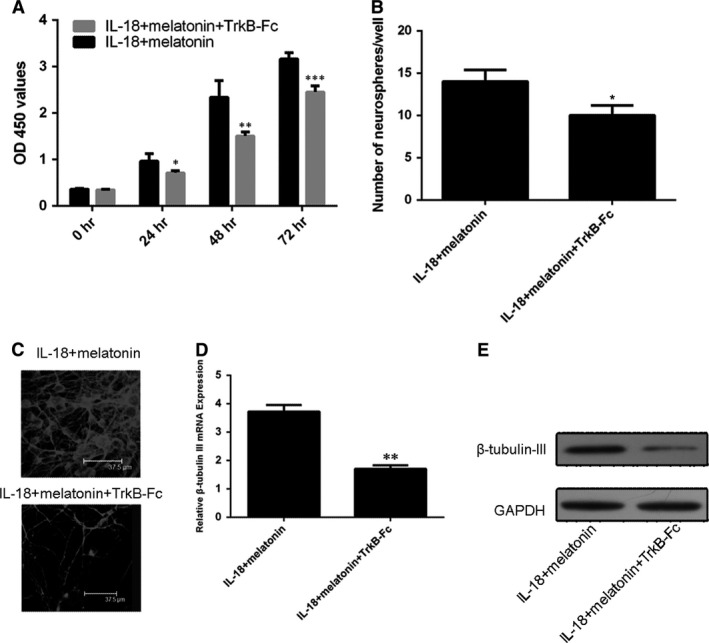
Effects of TrkB‐Fcon protective actions of melatonin in IL‐18‐challenged NSCs. (**A**) The proliferation profiles of NSCs treated with IL‐18 and/or melatonin (10 ng/ml) in the absence or presence of TrkB‐Fc. (**B**) The profiles of neurosphere formation. (**C**) Immunofluorescence staining for profiles of β‐tubulin‐III‐positive neurons derived from NSCs. (**D**) mRNA and (**E**) protein levels of β‐tubulin‐III, respectively. *P < 0.05; **P < 0.01 and ***P < 0.001.

**Figure 7:**
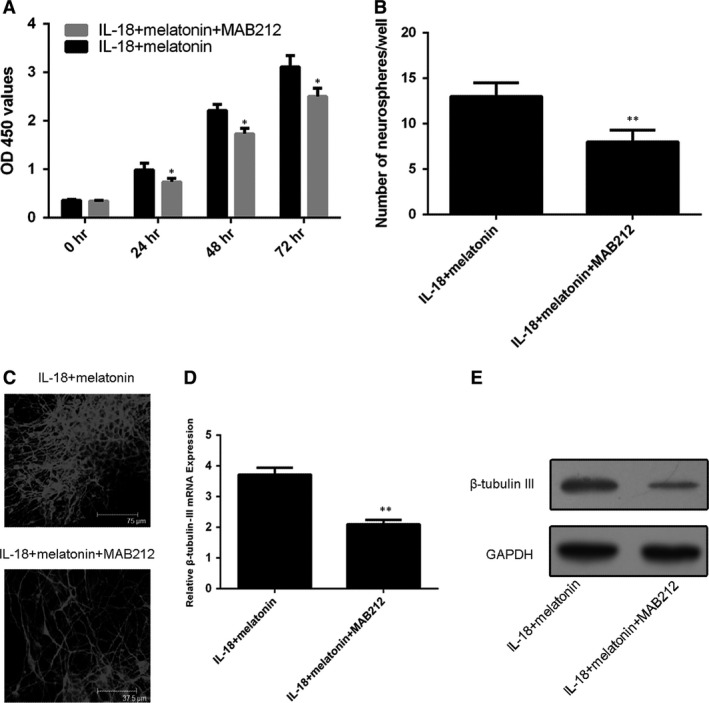
Effects of MAB212 on protective actions of melatonin in IL‐18‐challenged NSCs. (**A**) The proliferation profiles of NSCs treated with IL‐18 and/or melatonin (10 ng/ml) in the absence or presence of MAB212. (**B**) MAB212 blocked the promoting part effect of melatonin on the profiles of neurosphere formation. (**C**) Immunofluorescence staining for β‐tubulin‐III‐positive neurons derived from NSCs. (**D**) mRNA and (**E**) protein levels of β‐tubulin‐III, respectively, *P < 0.05; **P < 0.01.

It was observed that both BDNF inhibitor (TrkB‐Fc) and GDNF inhibitor (MAB212) also partially reduced the beneficial effects of melatonin on the NSC proliferation and neurosphere formation under the IL‐18 challenging (Figs 6A,B and 7A,B). The results suggested that the BDNF and GDNF were the downstream factors of the melatonin's protective pathway on NSCs. Both TrkB‐Fc and MAB212 also abolished the promoting effect of melatonin on β‐tubulin‐III positivity (Figs 6C and 7C) as well as the expression of β‐tubulin‐III mRNA (Figs 6D and 7D) and protein (Figs 6E and 7E) in IL‐18‐challenged NSCs.

## Discussion

NSCs are immature, self‐renewing and undifferentiated precursor cells with an potential to differentiate to astrocytes, neurons or oligodendrocytes 37, 38. NSC transplantation could have therapeutic applications in various neuro‐pathological conditions, such as Alzheimer's disease, Parkinson's disease and spinal cord injury 39. However, several shortcomings including the retarded proliferation of the transplanted NSCs as well as the unwanted differentiation of the majority of NSCs into astrocytes hindered their clinical utilization 40. These problems may be in part due to the biological actions of several pro‐inflammatory cytokines, such as TNF‐α, IL‐1β, IL‐6, IL‐18, nitric oxide (NO). These pro‐inflammatory cytokines induce apoptosis, axonal destruction and extensive demyelination 41. Among these cytokines, IL‐18 plays a pivotal role for pathophysiological actions of NSCs 28. In the current study, we found that IL‐18 suppressed the proliferation and differentiation of rat NSCs in the cultured condition. To overcome the negative impacts of IL‐18 on NSAs melatonin was selected for this purpose. Melatonin is a naturally occurring molecule with the potent antioxidant and anti‐inflammatory activities 42, 43, 44, 45. Many studies have proved that melatonin reduces oxidative stress and inflammation in a variety of biological systems 18, 44, 46, 47, 48. These activities of melatonin are believed to be mediated by its direct free radical scavenging action and also *via* its induction on antioxidant and anti‐inflammatory enzymes 49, 50, 51. As a result, melatonin exerted significant neuroprotective effects in Alzheimer's disease, Parkinson's disease, ischaemic brain injury and spinal cord injury 52, 53. Kong *et al*. 35 reported that melatonin promoted the viability of cultured ventral midbrain‐derived NSCs, facilitated roxinehydroxylase‐positive neuronal differentiation and inhibited glial differentiation. Fu *et al*. 54 also found that melatonin produced beneficial effects as a supplement for treating neonatal hypoxic–ischaemic brain injury by promoting the proliferation and differentiation of NSCs. In addition, the melatonin has been shown to act as a protective mediator in lippolysaccharide‐challenged NSCs 55. In another stem cell study, Liu *et al*. 56 showed that melatonin maintained mesenchymal stem cell survival and promoted their osteogenic differentiation in inflammatory environment induced by IL‐1β. Consistent with these observations, we observed that IL‐18 inhibited NSC proliferation, neurosphere formation and their differentiation into neurons in cultured rat NSCs and all these negative effects mediated by IL‐18 were significantly reduced by melatonin supplementation. The mechanistic studies indicated that melatonin up‐regulation the gene expressions of both BDNF and GDNF. BDNF and GDNF are two major neurotrophic factors and they are essential for the NSC proliferation and differentiation in normal conditions 57, 58, 59. Both BDNF and GDNF can be considered as the downstream elements of melatonin's protective pathway for NSCs regarding their survival, proliferation and differentiation. It appears that this pathway is initiated by melatonin membrane receptors (MT1 and/or MT2) as the luzindole, a MT1/MT2 antagonist, significantly reduces the protective effects of melatonin on NSCs. Currently, few molecules have been identified to promote the proliferation and differentiation of NSCs. Our results strongly suggest that melatonin administration would increase the survival chance in NSC transplant therapy by promoting the proliferation of these cells. Most importantly, melatonin has the capacity to direct the NSCs differentiation into the neural cells but not the astrocytes and thus can generate the functional neurons after the NSC transplant.

The results identified the molecular mechanisms as to how melatonin would increase the survival rate and differentiation of NSCs and provided novel evidence for clinical application of melatonin as a neuroprotective agent in neuroinflammatory diseases, especially, considering the low or non‐toxicity of this molecule.

## Conflict of interest

The authors declare no conflict of interest.

## Author contributions

Z. L, X.Y L, X. Y, J.X. S set up the idea for writing the manuscript; Z. L, X. Y, J.X. S. collected the data regarding the manuscript; Z. L, X. Y, J.X. S analysed the data; Z. L, X. Y, J.X. S. wrote the original manuscript in English; and M.T.V. C, W. K. K. W., D. X. T revised the manuscript, worked on the English and made the final version of the manuscript. All authors reviewed the final version of manuscript.
